# Molecular-weight fractionation, preliminary structural characterization, bioactivity, and ICP-MS-based biodistribution of *Grifola frondosa* polysaccharide-derived fractions

**DOI:** 10.3389/fnut.2026.1930891

**Published:** 2026-08-27

**Authors:** Yao Sun, Wei Zheng, Xianling Yang, Jiayu Deng, Qipin Gao, Duoduo Xu, Yang Gao

**Affiliations:** 1 Department of Pharmacy, China-Japan Union Hospital of Jilin University, Changchun, China; 2 Jilin Ginseng Academy, Changchun University of Chinese Medicine, Changchun, China; 3 Departments of Ophthalmology, The Affiliated Hospital, Changchun University of Chinese Medicine, Changchun, China; 4 Chinese Traditional Medicine Division, Jilin Institute of Food and Drug Control, Changchun, China; 5 Department of Pharmacy, Lequn Branch, The First Hospital of Jilin University, Changchun, China; 6 Country School of Pharmacy, Changchun University of Chinese Medicine, Changchun, China

**Keywords:** biodistribution, functional foods, grifola frondosa polysaccharides, ICP-MS, immunostimulation, molecular-weight fractionation, polysaccharide-derived fractions, preliminary structural characterization

## Abstract

**Introduction:**

*Grifola frondosa* is an edible and medicinal mushroom rich in bioactive polysaccharides, but the relationships among molecular-weight distribution, compositional and linkage features, biological activity, and *in vivo* distribution remain incompletely characterized.

**Methods:**

Polysaccharides from *G. frondosa* were extracted, purified, and separated into three molecular-weight fractions: PP-1 (>10 kDa), PP-2 (1-10 kDa), and PP-3 (< 1 kDa). The fractions were characterized by total sugar, acidic sugar and protein contents, monosaccharide composition, apparent molecular-weight distribution, and methylation analysis. Their preliminary bioactivities were evaluated using S180, HepG2, and RAW 264.7 cell-based assays. PP-3 was subsequently labeled with fluorescein isothiocyanate followed by iodination, and iodine content was quantified using inductively coupled plasma mass spectrometry (ICP-MS) to evaluate its preliminary *in vivo* distribution.

**Results:**

Among the three fractions, PP-3 showed the highest sugar content and a relatively simple detected monosaccharide profile under the present Glc/Gal-standard-based analysis, with methylation analysis suggesting abundant terminal Glcp residues and limited internal glucose linkages. PP-3 exhibited the strongest preliminary *in vitro* antiproliferative activity against S180 sarcoma and HepG2 hepatocellular carcinoma cells under the present screening conditions. It also enhanced RAW 264.7 macrophage proliferation and increased nitric oxide, TNF-α, IL-1β, and IL-6 production. The ICP-MS method showed good linearity, precision, stability, reproducibility, and recovery. After oral administration in rats, iodine-labeled PP-3-derived material showed limited systemic absorption, with the highest blood absorption rate observed at 2 h, and iodine signal was mainly detected in the liver and kidney.

**Discussion:**

Because ICP-MS detects iodine rather than the intact carbohydrate backbone, the biodistribution findings should be interpreted as preliminary tracer-based distribution data for iodine-labeled PP-3-derived material rather than a full pharmacokinetic profile of the native PP-3 fraction. Overall, this study identifies PP-3 as a low-molecular-weight carbohydrate/oligosaccharide-rich fraction with the strongest preliminary *in vitro* bioactivity among the tested *G. frondosa* fractions and provides an ICP-MS-based strategy for tracing iodine-labeled PP-3-derived material, supporting further investigation of mushroom-derived polysaccharide-derived fractions for functional-food and natural-product applications.

## 1 Introduction

Edible and medicinal mushrooms are important natural sources of nutrients and bioactive compounds. Recent reviews have further emphasized that edible mushrooms contain diverse bioactive compounds, including polysaccharides, proteins, phenolic compounds, sterols, and terpenoids, with potential immunomodulatory, antioxidant, anticancer, hepatoprotective, and metabolic activities (1, 2). However, the development of mushroom-derived bioactive ingredients remains limited by incomplete structural characterization, variability in extraction and purification procedures, limited standardization, uncertain bioavailability, and insufficient translational evidence (2). Their fruiting bodies contain proteins, minerals, vitamins, and diverse secondary metabolites, while generally being low in fat (3, 4). Among mushroom-derived constituents, polysaccharides, particularly β-glucans and related glucans, have attracted sustained attention because of their immunomodulatory, antioxidant, antimicrobial, anti-inflammatory, and antiproliferative activities (5–12). These properties make mushroom polysaccharides promising candidates for functional-food development and natural-product research. However, their biological activities are strongly influenced by structural features, including molecular weight, monosaccharide composition, glycosidic linkage pattern, branching degree, and solubility.

*Grifola frondosa*, commonly known as Maitake or “hen-of-the-woods,” is an edible and medicinal mushroom distributed in temperate regions of Asia, Europe, and North America (13, 14). In traditional East Asian medicine and modern functional-food research, *G. frondosa* has been valued for its polysaccharide-rich extracts. Previous studies have shown that *G. frondosa* polysaccharides can stimulate macrophages, T cells, and natural killer cells and may exert immunomodulatory, antioxidant, antiviral, metabolism-related, and antiproliferative effects (15–21). More recently, Ding et al. (22) reported the structural characterization of *G. frondosa* polysaccharide and its effects on insulin resistance in high-fat-diet-fed mice, further supporting the biological relevance of *G. frondosa* polysaccharides while highlighting the importance of detailed structural characterization. Nevertheless, many previous studies have used crude or partially purified extracts, making it difficult to clarify how specific molecular-weight fractions and structural characteristics contribute to biological activity.

Polysaccharides are structurally heterogeneous macromolecules, and this heterogeneity complicates their purification, characterization, activity comparison, and *in vivo* tracking. Molecular-weight distribution is particularly important because it can influence solubility, receptor interaction, intestinal absorption, tissue distribution, and biological response. In addition, the *in vivo* behavior of natural polysaccharides remains difficult to evaluate. Conventional approaches such as chromatography, isotope labeling, fluorescence tracing, and immunoassays each have limitations, including limited specificity, potential structural alteration, fluorescence quenching or background interference, and safety concerns related to radioactive labeling. Therefore, more sensitive and practical analytical strategies are needed to investigate the biodistribution of structurally characterized polysaccharide fractions (23, 24).

In this study, we extracted and purified polysaccharides from *G. frondosa* and separated them into three molecular-weight fractions: PP-1 (>10 kDa), PP-2 (1–10 kDa), and PP-3 (< 1 kDa). We then systematically characterized their sugar, acidic sugar, and protein contents, monosaccharide composition, molecular-weight distribution, and glycosidic linkage patterns. Their biological activities were evaluated using tumor-cell and macrophage-based assays, and the most active fraction was further investigated *in vivo* using fluorescein isothiocyanate-assisted iodination coupled with inductively coupled plasma mass spectrometry. By integrating molecular-weight fractionation, preliminary structural characterization, cell-based bioactivity assays, and ICP-MS-based biodistribution analysis, this study aimed to explore preliminary associations among molecular-weight distribution, compositional/linkage features, cell-based bioactivity, and labeled-material distribution of *G. frondosa* polysaccharide-derived fractions, thereby supporting their further development as mushroom-derived natural products for functional-food and related applications.

## 2 Materials and methods

### 2.1 Materials and chemicals

**Figure 1:**
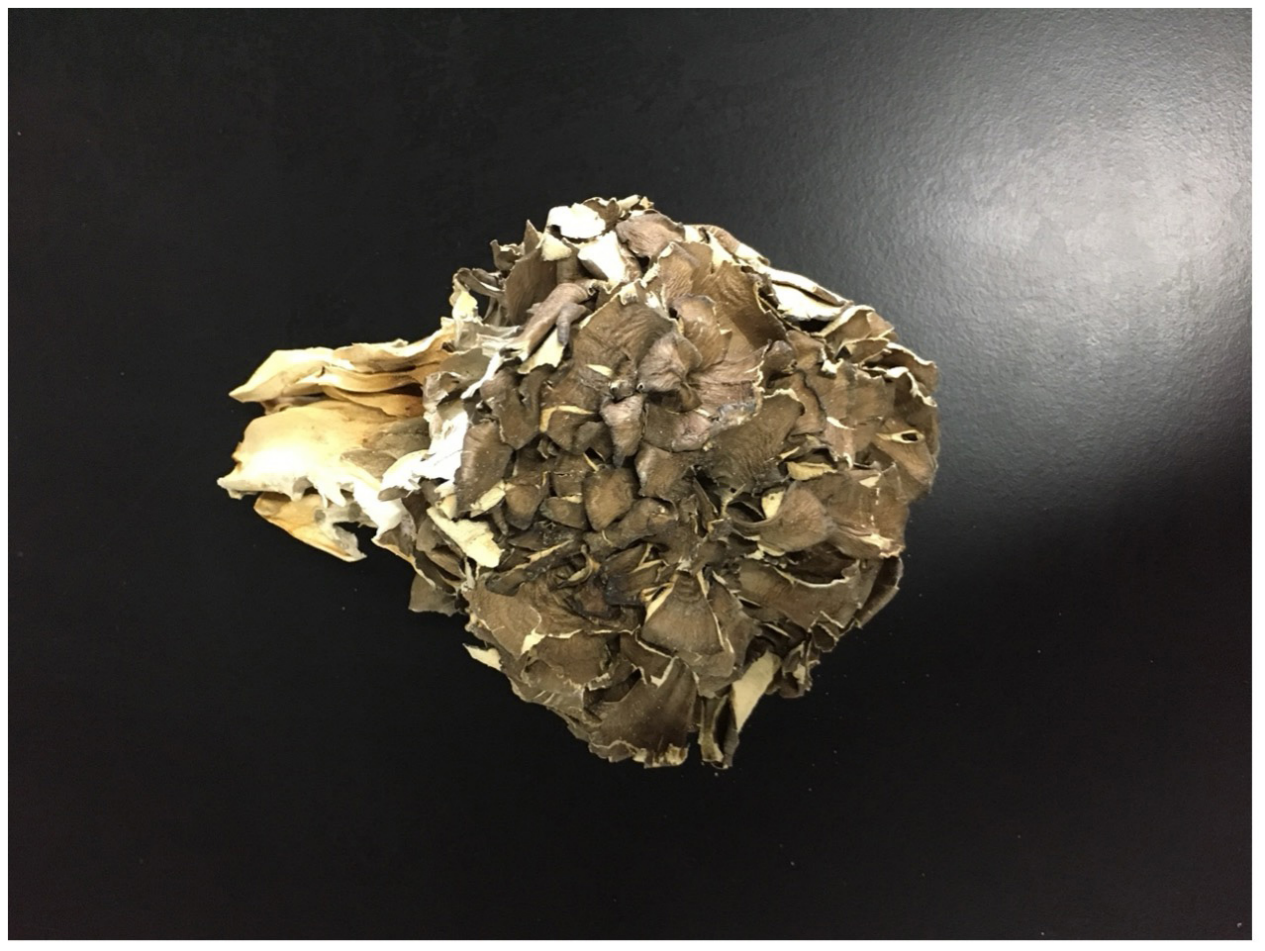
Fruiting body of *Grifola frondosa* used for polysaccharide extraction.

Fruiting bodies of *Grifola frondosa* were obtained from Fujian Province, China, and their authenticity was confirmed by Prof. Qipin Gao (Changchun University of Traditional Chinese Medicine) using macroscopic and microscopic taxonomic characteristics (Figure 1). Ultrafiltration membranes with molecular weight cut-offs of 10 kDa and 1 kDa were purchased from Mosutech (Shanghai, China). Ion exchange resins, including 717 and 732, were sourced from Shanghai Resin Factory (Shanghai, China). Standard molecular weight markers, such as T-dextran, glucose, and galactose, along with fluorescein isothiocyanate (FITC), cytokine assay kits for nitric oxide (NO), tumor necrosis factor-α (TNF-α), interleukin (IL)-1β, and IL-6, and a protein assay kit, were supplied by Sigma-Aldrich (St. Louis, MO, USA). The RAW 264.7 cell line (mouse macrophages), HepG2 cell line (human liver cancer cells), and S180 cell line (mouse sarcoma cells) were obtained from ATCC (Manassas, VA, USA). Additional analytical reagents were acquired from Beijing Chemical Works (Beijing, China) and Aladdin Bio-Chem Technology (Shanghai, China).

### 2.2 Extraction, purification, and separation of polysaccharides

**Figure 2:**
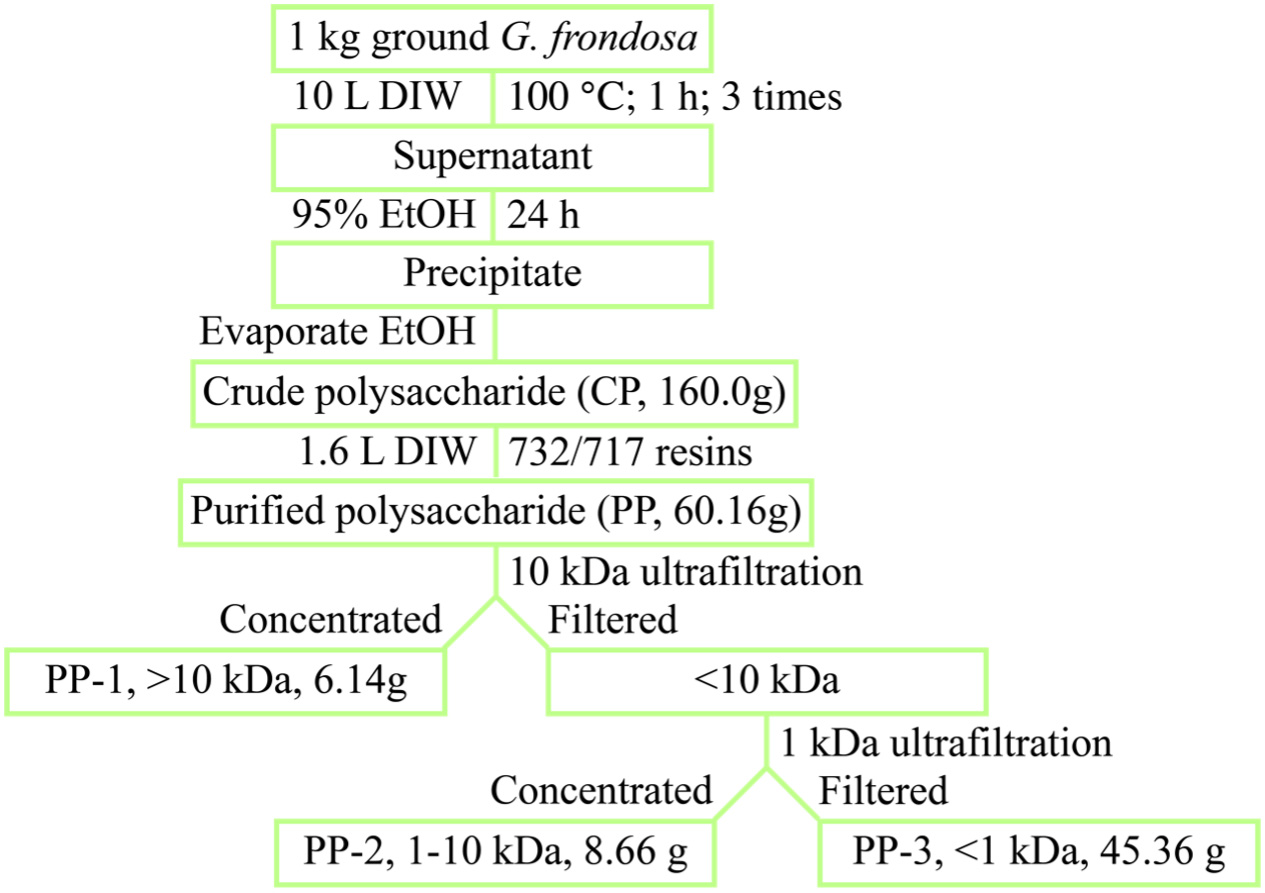
Schematic overview of the extraction, purification, and fractionation process of *Grifola frondosa* polysaccharides.

The process for extracting and purifying *G. frondosa* polysaccharides is outlined in Figure 2. Briefly, 1 kg of *G. frondosa* was ground and pre-soaked in 200 mL of water for 30 min to hydrate the material, facilitating efficient polysaccharide extraction. The hydrated material was then mixed with 10 L of deionized water (DIW) at a 1:10 (w/v) ratio and subjected to three rounds of decoction at 100 °C for 1 h each. The supernatant was collected and concentrated to 1 L, followed by the addition of 95% ethanol to achieve an 80% final ethanol concentration. After 24 h, the precipitate was collected, washed with 95% ethanol to remove impurities, and dried under vacuum. The final dry weight of the crude polysaccharide (CP) was 160.0 g.

CP was dissolved in DIW and passed through a mixed ion exchange resin column (10 × 100 cm) composed of a 1:1 weight ratio of 732 (cation exchange) and 717 (anion exchange) resins to remove salt and protein impurities. Deionized water (DIW) was used as the eluent to ensure effective purification. The eluate was collected and lyophilized, producing 60.16 g of purified polysaccharide (PP).

PP was further separated by ultrafiltration using membranes with molecular weight cut-offs of 10 kDa and 1 kDa, yielding three fractions: PP-1 (>10 kDa, 6.14 g), PP-2 (1–10 kDa, 8.66 g), and PP-3 (< 1 kDa, 45.36 g). These values represent the dry weights recovered after ultrafiltration and lyophilization. All polysaccharide fractions used in this study were prepared from a single authenticated bulk lot of *G. frondosa* and processed under identical conditions to minimize experimental variability.

### 2.3 Physicochemical properties of polysaccharides

#### 2.3.1 Assay of total sugar, acidic sugar, and protein

The content of total sugar, acidic sugar, and protein in the CP and its fractions is presented in Table 1. Total sugar was measured using the phenol-sulfuric acid method, acidic sugar was determined by the 3-Phenylphenol method, and protein content was assessed using the Bradford assay, as previously described in the literature (23, 25).

**Table 1:** Total sugar, acidic sugar, and protein content in *G. frondosa* polysaccharide fractions.

Sample	Total sugar (%)	Acidic sugar (%)	Protein (%)
CP	43.68 ± 0.56	36.37 ± 0.38	33.80 ± 1.72
PP	74.91 ± 0.28	17.97 ± 1.00	23.20 ± 0.33
PP-1	53.46 ± 0.41	17.91 ± 0.21	45.66 ± 1.10
PP-2	56.61 ± 0.55	51.22 ± 1.01	9.83 ± 0.41
PP-3	79.08 ± 0.48	10.82 ± 0.29	15.72 ± 0.87

#### 2.3.2 Monosaccharide composition analysis

The monosaccharide composition of PP-1, −2, and −3 was analyzed via pre-column derivatization. Samples (5 mg each) were hydrolyzed in 2 M trifluoroacetic acid (TFA) at 100 °C for 8 h, then dried with methanol. The residues were reacted with sodium hydroxide and 3-Methyl-1-phenyl-2-pyrazoline-5-one (PMP), then neutralized and extracted. The aqueous phase was analyzed using an Agilent ZORBAX Eclipse Plus C18 column (4.6 × 250 mm, 5 μm particle size) with detection at 250 nm. The mobile phase consisted of acetonitrile and 0.1 M potassium phosphate buffer (85:15, v/v), and the flow rate was set at 1.0 mL/min (23).

#### 2.3.3 Molecular weight distribution analysis

Molecular weight distribution was determined by gel permeation chromatography (GPC) using an Agilent HPLC system equipped with a refractive index detector and a Shodex OHpak SB-806M HQ column (8 × 300 mm, Showa Denko K.K., Japan). The mobile phase was 0.7% sodium sulfate solution, and the flow rate was 0.5 mL/min at 30 °C. Molecular weights were calculated based on a calibration curve generated using standard dextran samples (MW range: 1–70 kDa) (25). Because the calibration standards used in this study covered the 1–70 kDa range, molecular-weight estimates below 1 kDa were interpreted cautiously as apparent molecular weights rather than exact absolute molecular masses.

#### 2.3.4 Methylation analysis

Methylation analysis was performed on PP-1, PP-2, and PP-3 according to the Ciucanu method with minor modifications. Briefly, dried samples were dissolved in anhydrous dimethyl sulfoxide (DMSO), followed by addition of sodium hydroxide and iodomethane for methylation. The fully methylated products were then hydrolyzed with trifluoroacetic acid (TFA), reduced, and acetylated to generate partially methylated alditol acetates. The resulting derivatives were analyzed by gas chromatography–mass spectrometry (GC-MS). Linkage assignments were made by comparing retention times and mass-fragmentation patterns with published reference data.

### 2.4 Antiproliferative and immunostimulatory assays

#### 2.4.1 Cell culture

S180 cells were cultured in Roswell Park Memorial Institute 1640 medium (RPMI 1640), whereas HepG2 and RAW 264.7 cells were cultured in Dulbecco's modified Eagle's medium (DMEM). Both media were supplemented with 10% fetal bovine serum and 1% penicillin–streptomycin, and cells were maintained at 37 °C in a humidified incubator containing 5% CO_2_ (26–28).

#### 2.4.2 Cell viability assay

The CCK-8 assay was selected as a standard colorimetric cell-viability assay for preliminary evaluation of the antiproliferative effects of natural-product or polysaccharide-derived fractions in tumor cells (26–28). To evaluate antiproliferative activity, HepG2 or S180 cells were seeded into 96-well plates at 1 × 10^4^ cells/well and incubated for 24 h. The culture medium was then replaced with fresh medium containing *G. frondosa* polysaccharide fractions at concentrations ranging from 200 to 1,000 μg/mL. After 24 h of treatment, 10 μL of CCK-8 solution was added to each well, followed by incubation for 4 h at 37 °C. Absorbance was measured using a microplate reader, and the inhibition rate was calculated as follows:


$$\begin{array}{l} Inhibition\;Rate\;\left(\text{\%}\right)=\left(1-\frac{{OD}_{sample}-{OD}_{blank}}{{OD}_{control}-{OD}_{blank}}\right)\times \text{100}\% \end{array}$$

The CCK-8 cell-viability assay was performed with *n* = 3 independent replicates per group.

#### 2.4.3 Immunostimulatory effects

To evaluate the immunostimulatory effects, 200 μL of RAW 264.7 cells (1 × 10^4^ cells/well) were initially seeded in a 96-well plate and incubated for 24 h. The culture medium was then replaced with 200 μL of fresh medium, which either contained or did not contain *G. frondosa* polysaccharide at concentrations ranging from 50 μg/mL to 1,000 μg/mL. After an additional 24 h of incubation, the media in each well were collected and centrifuged. The levels of cytokines (TNF-α, IL-1β, and IL-6) and NO were measured using specific assay kits. Macrophage proliferation assays were performed with *n* = 3 independent replicates per group, and NO/cytokine measurements were performed with *n* = 6 independent samples per group.

### 2.5 ICP-MS-based *in vivo* biodistribution of PP-3

#### 2.5.1 FITC labeling reaction

FITC labeling of *G. frondosa* polysaccharide was performed following the Belder method (23, 25, 29). Briefly, 1.0 g of the active *G. frondosa* polysaccharide fraction, PP-3 was dissolved in 10 mL of DMSO, with the addition of 5 drops of pyridine under sonication. To this mixture, 0.1 g of FITC and 20 mg of dibutyltin dilaurate (DBTLD) were added, and the reaction was heated to 95 °C for 2 h. After cooling to room temperature, 95% ethanol (v/v) was added to achieve a final ethanol concentration of 85% (v/v) to induce precipitation of the FITC-labeled product. The resulting precipitate was collected, washed with ethanol, and subsequently dried. The final product, FITC-labeled *G. frondosa* polysaccharide (PP-F), was weighed and stored for further use.

#### 2.5.2 Iodination reaction

The iodination reaction was performed following the Keck method, with modifications based on our prior research (23, 30). Briefly, 1.0 g of dry PP-F was dissolved in 5 mL of DIW using sonication. To this solution, 2 mL of 0.5 M sodium iodide (NaI) and 5 mL of 0.1 g/mL Chloramine-T buffer solution were added. The mixture was vortexed for 1 min to ensure thorough mixing. Subsequently, 5 mL of 0.1 g/mL sodium metabisulfite buffer solution and 5 mL of a 0.1 g/mL potassium iodide (KI) solution were added, followed by shaking.

#### 2.5.3 Purification of the iodine-substituted polysaccharide

Starting with the supernatant containing the iodine-substituted polysaccharide from the previous step, the purification procedure can be summarized as follows: The supernatant was separated using a Sephadex G-10 column (50 cm × 5 cm) via medium-pressure preparative liquid chromatography (CHEETAH™ MP, Agela, Tianjin, China). The column was eluted with DIW. The fraction containing the polysaccharide was identified using carbohydrate colorimetric detection and electrical conductivity measurements. The purified iodinated polysaccharide (PP-FI) was obtained by collecting the relevant fraction, concentrating it, and then freeze-drying to yield the final product.

#### 2.5.4 Determination of iodination rate of iodinated polysaccharide by ICP-MS and method development

The iodination rate of the iodine-substituted polysaccharide was determined by ICP-MS according to a previously established method from our laboratory, with minor modifications (23). Before ICP-MS analysis, all samples were digested as described below. Specifically, 0.05 g of each sample was placed in a 10 mL headspace vial, followed by the addition of 4 mL of DIW, 3 mL of 25% tetramethylammonium hydroxide (TMAH), and 100 μL of hydrogen peroxide. The vial was tightly sealed, and digestion was performed for 30 min at 80 °C under sonication. Once cooled to room temperature (RT), the solution was transferred to a 25 mL volumetric flask, which was then diluted to the mark using 1% TMAH solution to ensure uniform mixing. A 0.1 mL aliquot of the diluted solution was further diluted in 25 mL of 1% TMAH, mixed thoroughly, filtered through a 0.22 μm nylon membrane, and finally subjected to ICP-MS analysis. A 1% TMAH solution was used as a negative control throughout the analysis.

The ICP-MS conditions were established based on previously validated protocols, summarized in Table 2 (23, 31). The tuning solution contained 7Li, 59Co, 89Y, 140Ce, and 205Tl, while a 1 mg/mL indium solution was used as an internal standard. To mitigate potential memory effects from prior sample injections, the ICP-MS system was flushed for 2 min between runs to ensure accurate results.

**Table 2:** ICP-MS measurement conditions for iodine detection in *Grifola frondosa* polysaccharides.

Instrument parameters	Reference value
Radio frequency power	1,550 W
Sampling cone/skimmer cone	1/0.4 mm
Sampling depth	8 mm
Nebulizer chamber temperature	2 °C
Collision reaction cell gas flow	5.0 mL/min
Internal standard addition mode	Inline Addition
Plasma flow rate	15 L/min
Dilution gas flow rate	0.60 L/min
Scanning mode	Peak Hopping
Carrier gas flow rate	0.74 L/min

To prepare the iodine standard solutions, NaI was used as the iodine source compound. All standard concentrations were expressed as iodine-equivalent concentrations after correction for the molecular-weight ratio of iodine to NaI. Specifically, iodine-equivalent concentrations were calculated using the conversion factor 126.90/149.89 = 0.8466. The iodine-equivalent standard working solutions were prepared with 1% TMAH at concentrations of 1, 10, 50, 100, 200, and 300 ng/mL and subjected to ICP-MS analysis to generate the iodine standard curve for quantification. Accordingly, iodine concentrations measured in PP-FI samples and biological samples were expressed as iodine-equivalent concentrations.

##### 2.5.4.1 Determination of ICP-MS precision

To assess the precision of ICP-MS, 0.05 g of PP-FI was accurately weighed and processed by digestion with deionized water, tetramethylammonium hydroxide (TMAH), and hydrogen peroxide, as described in Section 2.5.4.

##### 2.5.4.2 Determination of sample stability

The stability of the PP-FI samples was assessed by taking samples at 0, 2, 16, and 24 h post-preparation from the precision determination experiments. These samples were analyzed using ICP-MS, and the relative standard deviation (RSD) was calculated to evaluate the stability of the iodinated polysaccharide over time.

##### 2.5.4.3 Investigation of reproducibility

To assess reproducibility, six samples from the same batch of PP-FI (0.05 g each) were prepared and analyzed under identical conditions. The measurements were recorded, and the RSD was calculated to provide an indication of reproducibility in sample preparation and analysis.

##### 2.5.4.4 Determination of spike recovery rate

The recovery rate of iodine from PP-FI samples was evaluated by spiking six samples with varying concentrations of standard iodine solutions. Each spiked sample, containing 25 mg of PP-FI, was processed as described earlier and analyzed using ICP-MS. The recovery rate was calculated to assess the method's accuracy in detecting added iodine.

##### 2.5.4.5 Determination of independently prepared PP-FI samples

Three independently prepared PP-FI samples generated from separate iodination reactions were processed and analyzed by ICP-MS. The iodination rate was calculated using the following formula:


$$\begin{array}{l} Iodination\;Rate\;\left(\%\right)=\frac{M\times D}{G}\times \text{100}\% \end{array}$$

Note: M, Iodine content; D, Dilution factor; G, Sample weight.

##### 2.5.4.6 Investigation of the stability of PP-FI

To assess the stability of PP-FI in biological media, 40 mg of PP-FI was accurately weighed and dissolved in 20 mL of low-glucose DMEM culture medium. Three replicate samples were incubated at 37 °C in a constant-temperature shaking incubator. At intervals of 1, 2, and 3 h, 5 mL of each sample was collected, filtered through a 0.45 μm nylon filter membrane, and processed through a Sephadex G-10 medium-pressure preparative chromatography column (Φ 35 × 200 mm) with ultrapure water as the mobile phase. The eluent was combined, diluted, filtered, and analyzed as per the ICP-MS conditions outlined above to determine the stability of PP-FI.

#### 2.5.5 Determination of biodistribution of the polysaccharide

To investigate the biodistribution of PP-FI, twenty Wistar rats were acclimated in a standard environment for three days. The rats were randomly assigned into two groups: a control group and a PP-FI-treated group. After fasting for 24 h, each rat was orally administered either normal saline or PP-FI at 100 mg/100 g body weight. Small-volume blood samples were serially collected from the same rats at 1, 2, and 3 h after administration for iodine quantification. The sampling volume was minimized and did not exceed approximately 0.2 mL per rat per time point; therefore, the total serial blood-sampling volume did not exceed approximately 0.6 mL per rat before euthanasia. At 3 h after administration, rats were anesthetized by intraperitoneal injection of sodium pentobarbital at 50 mg/kg. After confirmation of deep anesthesia by the absence of pedal withdrawal reflex, rats were euthanized by CO_2_ inhalation using a gradual-fill method at a displacement rate of 30% of the chamber volume per minute. Death was confirmed by the absence of respiration and heartbeat, after which organs, including the liver, heart, spleen, lung, and kidney, were immediately harvested.

The harvested organs were washed with saline, blotted dry with filter paper, and freeze-dried to ensure consistency and preserve their structure for analysis. All animal procedures were approved by the Experimental Animal Ethics Committee of Changchun University of Chinese Medicine (Approval No. 2025530, dated 30 April 2025) and performed in accordance with national guidelines for the care and use of laboratory animals.

The blood and organ samples were digested following previously described methods, and iodine content was measured via ICP-MS. Iodine-equivalent signals measured in saline-treated control rats were used to estimate endogenous/background iodine signals. For blood samples, the mean iodine-equivalent concentration of the saline control group at each corresponding time point was subtracted from the value measured in the PP-FI-treated group. For organ samples, the mean iodine-equivalent signal of each corresponding organ in the saline control group was subtracted from that of the PP-FI-treated group. The PP-FI content in the blood and organs was calculated using the following formulas:


$$\begin{array}{l} C_{b}=\frac{M\times D}{IR}\times V\times {\text{10}}^{-6} \end{array}$$

$$AR\left(\%\right)=\frac{C_{b}(C_{o})}{AD}\times 100\%$$

Note: Cb, PP-FI content in the blood; *M*, Iodine content; *D*, Dilution factor; IR: Iodination rate; *V*, Rat blood volume (7.4% of body weight); Co, PP-FI content in organs; *W*, Organ weight; AR, Absorption rate; AD, Administration dose.

The *in vivo* biodistribution experiment included 20 rats, with *n* = 10 rats in the control group and *n* = 10 rats in the PP-FI-treated group.

### 2.6 Statistical analysis

All quantitative data are presented as mean ± standard deviation (SD). For cell-based assays, the number of independent replicates or samples is indicated in the corresponding figure legends. For CCK-8 cell-viability assays and RAW 264.7 macrophage-proliferation assays, data were obtained from three independent experiments. For cytokine/NO measurements, data were obtained from six independent samples. For ICP-MS method validation, replicate numbers are reported in the corresponding Supplementary Tables. For the *in vivo* biodistribution experiment, *n* = 10 rats per group were used unless otherwise indicated.

Group differences were analyzed using one-way analysis of variance (ANOVA) followed by Tukey's *post-hoc* test for multiple comparisons. For dose-response experiments, comparisons were performed among treatment groups under the same concentration or time point, as indicated in the corresponding figure legends. Statistical analysis was performed using Prism software (GraphPad Software, Inc., San Diego, CA, USA). A two-sided *p* value < 0.05 was considered statistically significant. Significance levels are indicated as ns, not significant; ^*^p < 0.05; ^**^p < 0.01, unless otherwise specified.

## 3 Results

### 3.1 Extraction, purification, and separation of *G. frondosa* polysaccharides

Crude polysaccharides (CP) were obtained from *G. frondosa* by water extraction and alcohol precipitation, yielding 160.0 g from 1 kg of starting material. After purification using ion exchange resin, 60.16 g of purified polysaccharides (PP) were obtained. PP was further separated by ultrafiltration into three molecular-weight fractions: PP-1 (>10 kDa, 6.14 g), PP-2 (1–10 kDa, 8.66 g), and PP-3 (< 1 kDa, 45.36 g). The corresponding recovered proportions of PP-1, PP-2, and PP-3 were 10.2%, 14.4%, and 75.4%, respectively.

### 3.2 Physicochemical properties of *G. frondosa* polysaccharide-derived fractions

The total sugar, acidic sugar, and protein contents of CP, PP, and the three PP fractions are presented in Table 1. After purification, the total sugar content increased from 43.68% in CP to 74.91% in PP, whereas the protein content decreased from 33.80% to 23.20%. Among the separated fractions, PP-3 had the highest total sugar content (79.08%) and a protein content of 15.72%. These results indicate that purification and ultrafiltration enriched carbohydrate components but did not yield completely protein-free or chemically homogeneous fractions. Therefore, the separated materials are described in this study as polysaccharide-derived fractions, and PP-3 is interpreted as a low-molecular-weight carbohydrate/oligosaccharide-rich fraction containing measurable residual protein.

**Figure 3:**
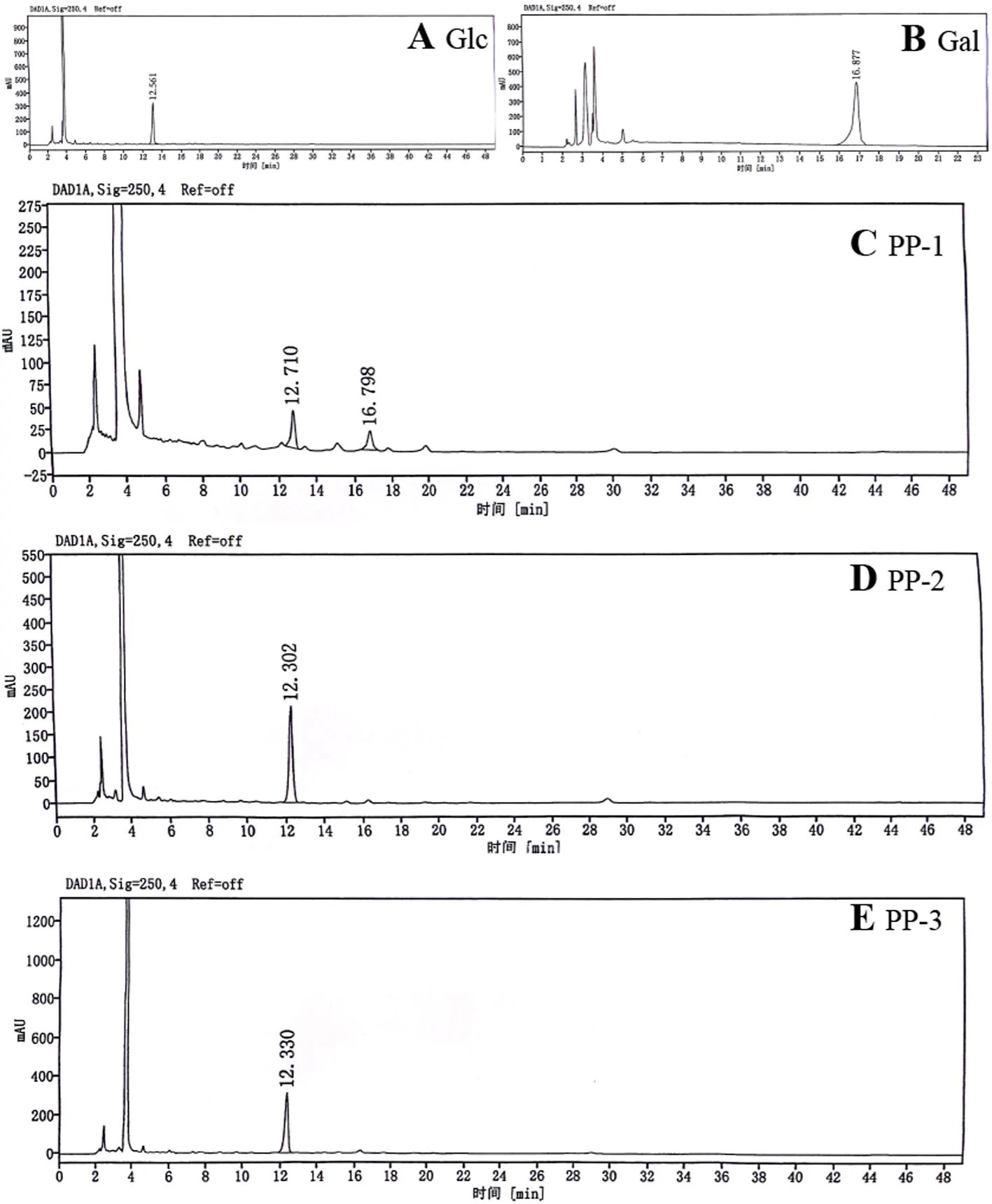
HPLC chromatograms of monosaccharide standards **(A)** Glucose (Glc) and **(B)** Galactose (Gal) and polysaccharide fractions **(C)** PP-1, **(D)** PP-2, and **(E)** PP-3.

Monosaccharide composition analysis was performed using glucose (Glc) and galactose (Gal) as standards (Figures 3A, B). PP-1 showed detectable Glc and Gal signals at a molar ratio of 1.5:1 under the present analytical conditions (Figure 3C). In contrast, among the two monosaccharide standards tested, only Glc was identified in PP-2 and PP-3 (Figures 3D, E). Because the monosaccharide analysis in this study used only Glc and Gal standards, the complete monosaccharide profiles of PP-2 and PP-3, particularly possible acidic sugars or uronic acid residues, could not be fully determined. Molecular-weight distribution was further analyzed by GPC, and the chromatographic profiles of PP-1, PP-2, and PP-3 are provided in Supplementary Figure S1. The apparent molecular weights of PP-1, PP-2, and PP-3 were estimated as 10,312 Da, 1,107 Da, and 184 Da, respectively. Because the apparent value obtained for PP-3 was below the lower range of the dextran calibration standards used in this study, this result should be interpreted cautiously as an apparent estimate rather than an exact absolute molecular weight. Accordingly, PP-3 is more appropriately described as a low-molecular-weight carbohydrate/oligosaccharide-rich fraction rather than a conventional high-molecular-weight polysaccharide.

### 3.3 Methylation analysis

**Figure 4:**
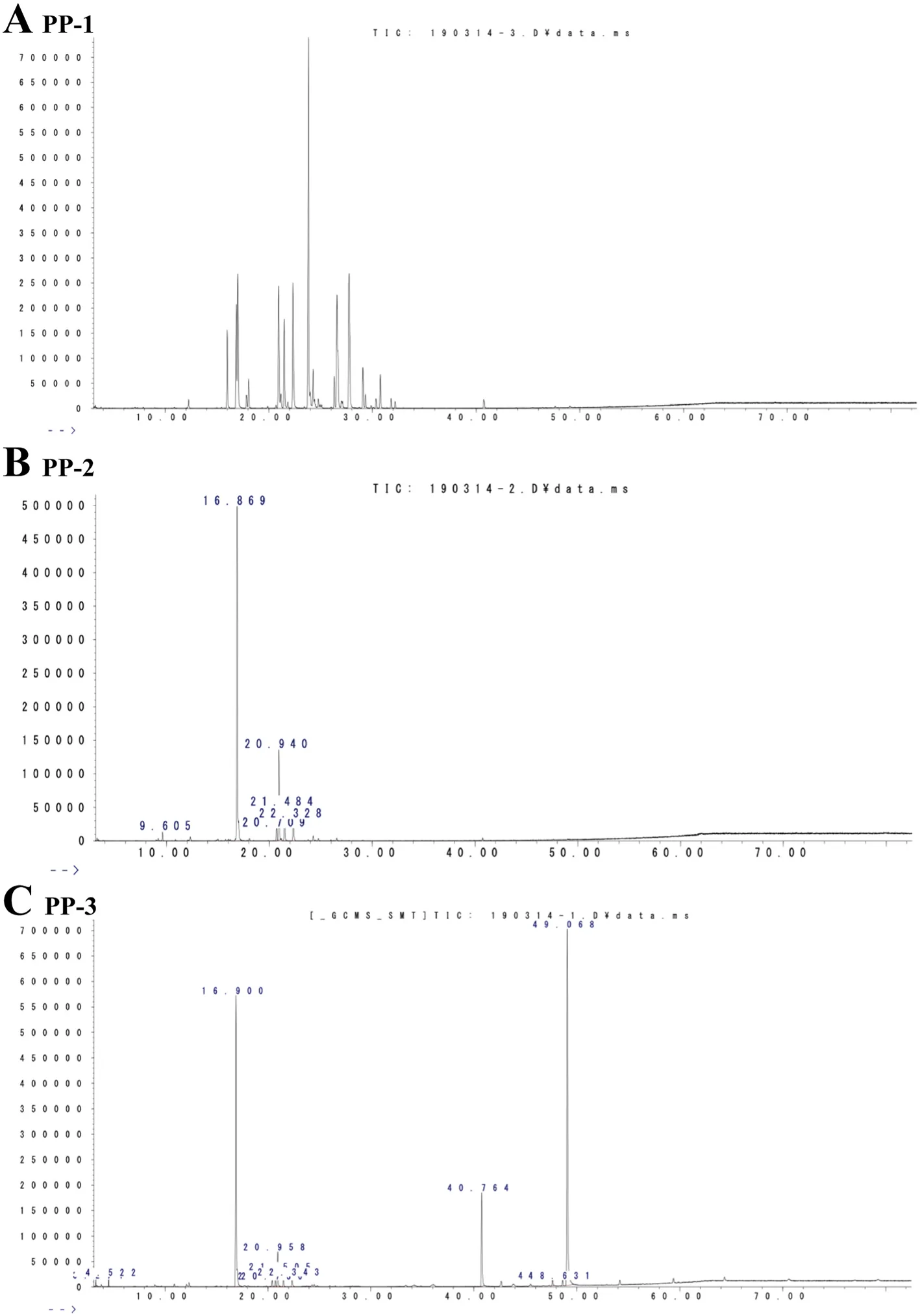
Total ion chromatograms (TIC) of methylated polysaccharide fractions **(A)** PP-1, **(B)** PP-2, and **(C)** PP-3.

**Figure 5:**
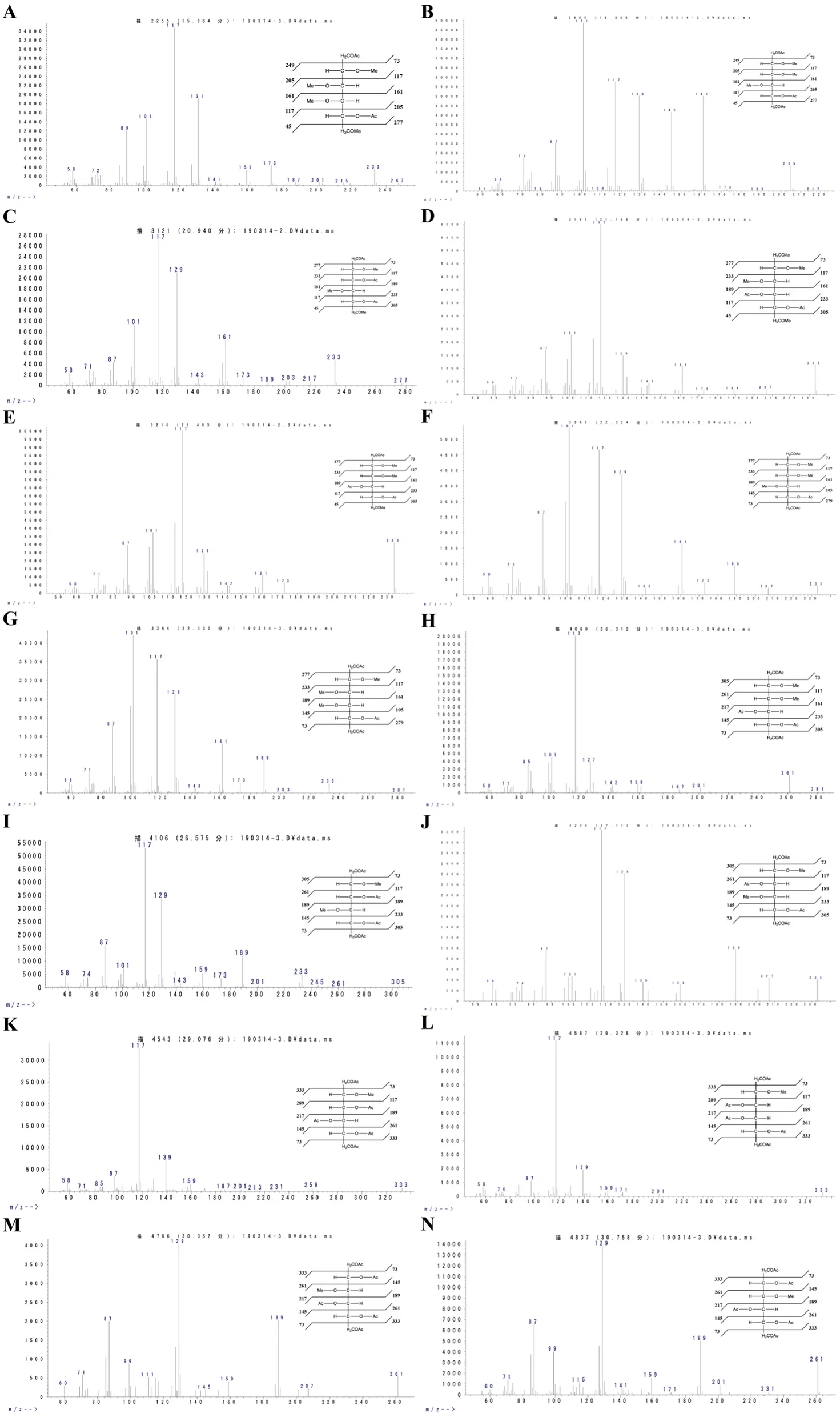
Mass spectra of methylated alditol acetate derivatives of polysaccharide fractions PP-1,−2, and−3. **(A)** 1, 5-Di-O-acetyl-2, 3, 4, 6-Tetra-O-methyl-Galactitol, **(B)** 1, 5-Di-O-acetyl-2, 3, 4, 6-Tetra-O-methyl-Glucitol, **(C)** 1, 3, 5-Tri-O-acetyl-2, 4, 6-Tri-O-methyl-Glucitol, **(D)** 1, 4, 5-Tri-O-acetyl-2, 3, 6-Tri-O-methyl-Galactitol, **(E)** 1, 4, 5-Tri-O-acetyl-2, 3, 6-Tri-O-methyl-Glucitol, **(F)** 1, 5, 6-Tri-O-acetyl-2, 3, 4-Tri-O-methyl-Glucitol, **(G)** 1, 5, 6-Tri-O-acetyl-2, 3, 4-Tri-O-methyl-Galactitol, **(H)** 1, 4, 5, 6-Tetra-O-acetyl-2, 3-Di-O-methyl-Glucitol, **(I)** 1, 3, 5, 6-Tetra-O-acetyl-2, 4-Di-O-methyl-Glucitol, **(J)** 1, 3, 5, 6-Tetra-O-acetyl-2, 4-Di-O-methyl-Galactitol, **(K)** 1, 3, 4, 5, 6-Penta-O-acetyl-2-O-methyl-Glucitol, **(L)** 1, 3, 4, 5, 6-Penta-O-acetyl-2-O-methyl-Galactitol, **(M)** 1, 2, 4, 5, 6-Penta-O-acetyl-3-O-methyl-Galactitol, **(N)** 1, 2, 4, 5, 6-Penta-O-acetyl-3-O-methyl-Glucitol. Structural formulas shown on the right side of each sub-figure represent the proposed sugar residues based on the methylation data. These structures are speculative and based on the analysis of methylated derivatives.

The total ion chromatograms of methylated PP-1, PP-2, and PP-3 are shown in Figure 4, and the mass spectra of methylated alditol acetate derivatives are shown in Figure 5. The methylation data are summarized in Table 3, with retention times compared against published reference values (32).

**Table 3:** Methylation Analysis of PP-1, PP-2, and PP-3.

Retention time (min)	Label in Figure 5	Methylated sugar	Deduced linkage pattern	Molar ratio		
				PP-1	PP-2	PP-3
15.9	A	2,3,4,6-Me4-Gal	T-Galp-(1 →	28		
16.9	B	2,3,4,6-Me4-Glc	T-Glcp-(1 →	18	15	26
20.9	C	2,4,6-Me3-Glc	→ 3)-Glcp-(1 →	18	3.5	3
21.1	D	2,3,6-Me3-Gal	→ 4)-Galp-(1 →	3		
21.5	E	2,3,6-Me3-Glc	→ 4)-Glcp-(1 →	13	1.5	1.5
22.3	F	2,3,4-Me3-Glc	→ 6)-Glcp-(1 →	50	1	1
22.8	G	2,3,4-Me3-Gal	→ 6)-Galp-(1 →	18		
26.3	H	2,3-Me2-Glc	→ 4,6)- Glcp-(1 →	4		
26.6	I	2,4-Me2-Glc	→ 3,6)- Glcp-(1 →	20		
27.1	J	2,4-Me2-Gal	→ 3,6)- Galp-(1 →	4		
29.1	K	2-Me-Glc	→ 3,4,6)- Glcp-(1 →	3		
29.3	L	2-Me-Gal	→ 3,4,6)- Galp-(1 →	4		
30.4	M	3-Me-Gal	→ 2,4,6)- Galp-(1 →	1		
30.8	N	3-Me-Glc	→ 2,4,6)- Glcp-(1 →	1		

PP-1 contained 14 types of sugar residues, including one terminal residue, three single-branch-point residues, and four double-branch-point residues composed of Glc and Gal. In contrast, PP-2 and PP-3 showed simpler methylation profiles, each containing three major detected glycosidic linkage types. Both PP-2 and PP-3 were mainly composed of glucose residues and showed relatively simple methylation profiles dominated by terminal Glcp residues, with lower amounts of internally linked Glcp residues. Among the detected internal glucose linkages, → 3)-Glcp-(1 → was more abundant than → 4)-Glcp-(1 → and → 6)-Glcp-(1 → in both PP-2 and PP-3. Because NMR spectroscopy was not performed, these linkage assignments should be interpreted as methylation-based preliminary structural information rather than definitive structural elucidation.

### 3.4 Antiproliferative activity

**Figure 6:**
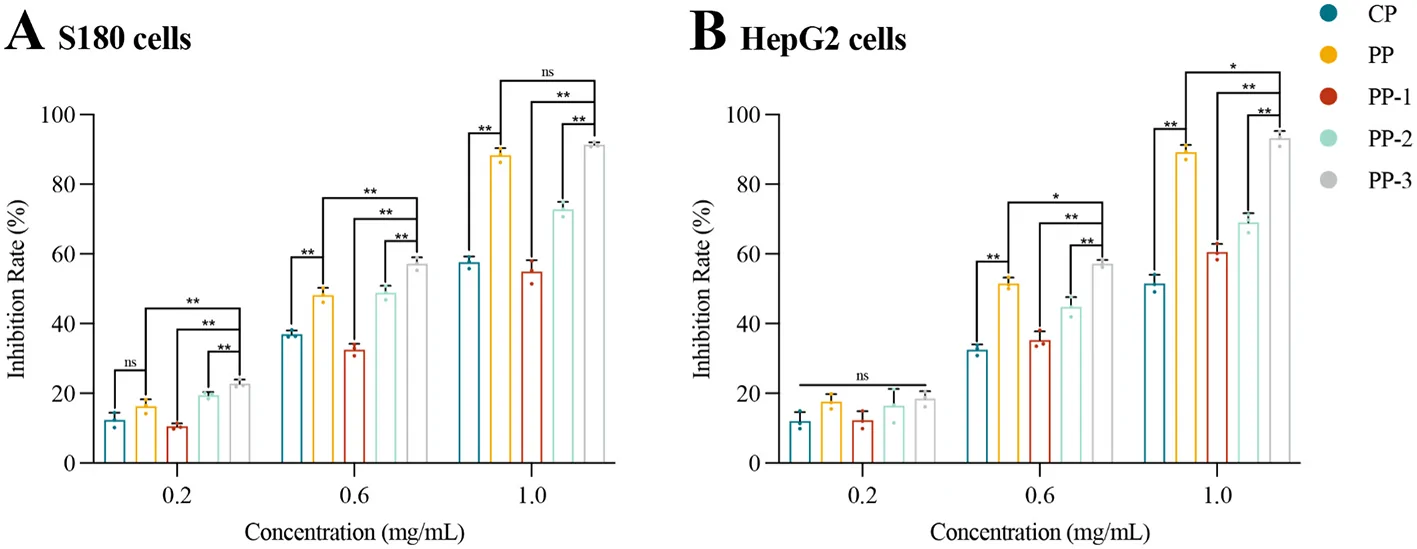
Dose-dependent inhibition of **(A)** S180 sarcoma and **(B)** HepG2 liver cancer cells by *Grifola frondosa* polysaccharide fractions (*n* = 3, mean ± SD, ns = not significant, *p < 0.05, **p < 0.01).

The antiproliferative effects of *G. frondosa* polysaccharide-derived fractions on S180 and HepG2 cells are shown in Figure 6. All tested polysaccharide samples showed concentration-dependent inhibitory effects, with the highest inhibition observed at 1,000 μg/mL. Among the purified fractions, PP-3 showed the strongest inhibitory activity against both S180 and HepG2 cells.

These findings indicate that PP-3 showed the strongest preliminary antiproliferative activity among the tested fractions under the present CCK-8 screening conditions. However, because positive antitumor controls and normal non-tumor cell controls were not included, the relative potency and cancer-cell selectivity of PP-3 could not be determined.

### 3.5 Immunostimulatory activity

**Figure 7:**
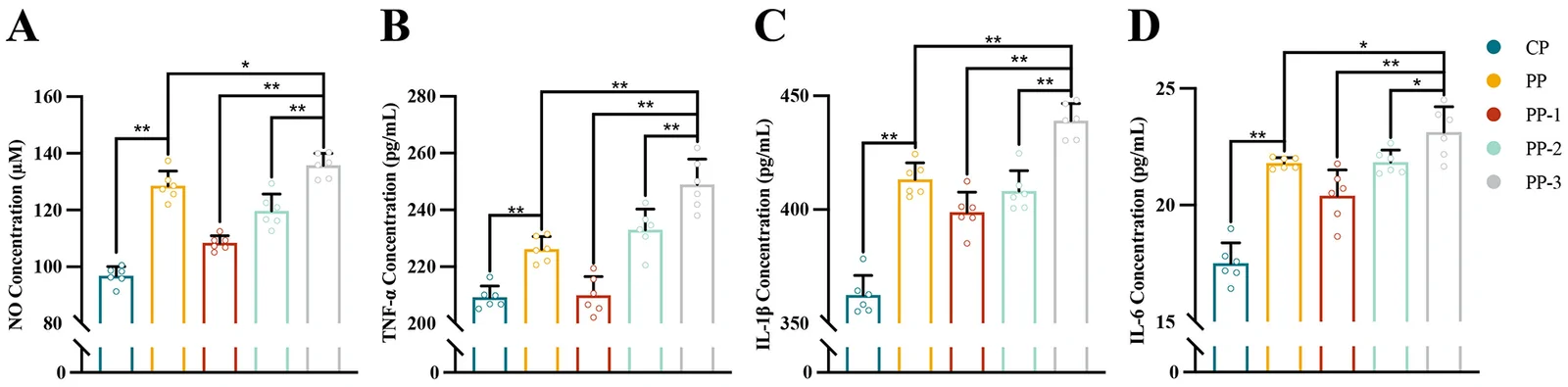
Immunostimulatory effects of *Grifola frondosa* polysaccharide fractions on RAW 264.7 macrophages at a concentration of 500 μg/mL: **(A)** NO, **(B)** TNF-α, **(C)** IL-1β, and **(D)** IL-6 concentrations measured using the corresponding assay kits (n = 6, mean ± SD, *p < 0.05, **p < 0.01).

**Figure 8:**
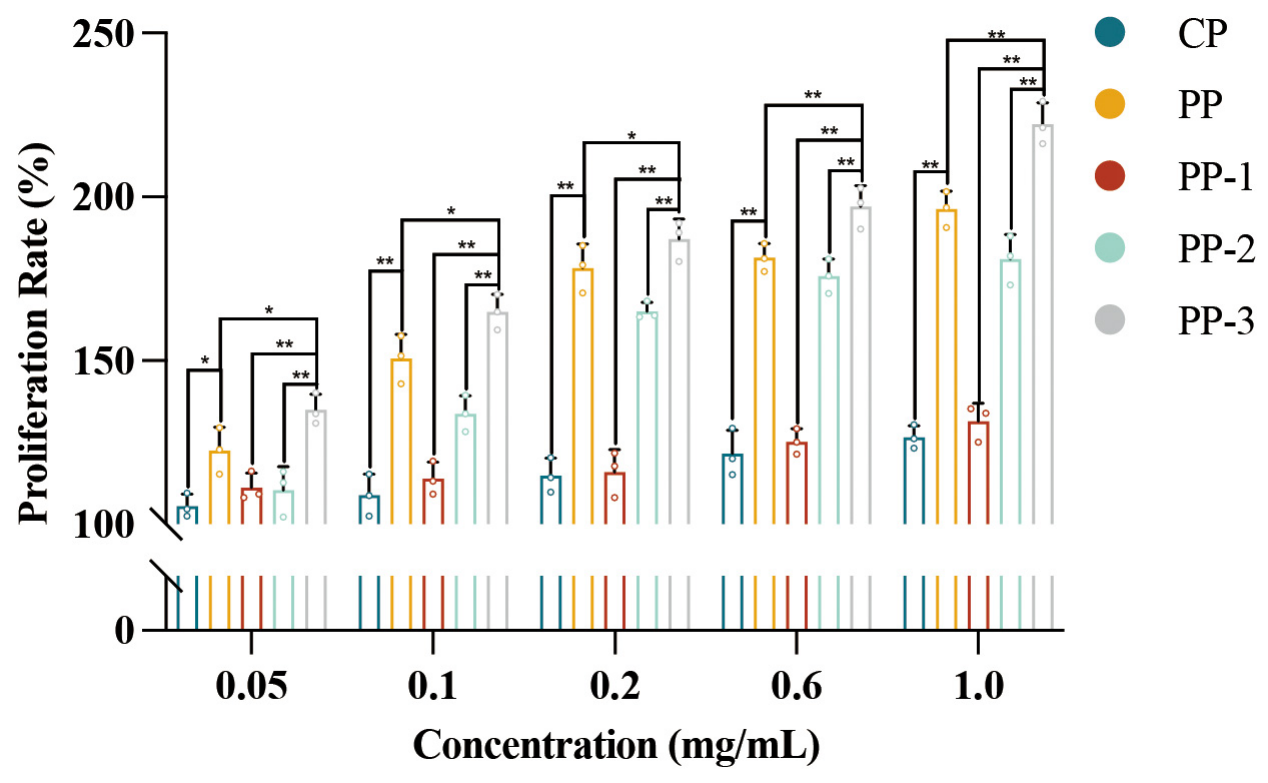
Dose-dependent proliferation of RAW 264.7 macrophages induced by *G. frondosa* polysaccharide fractions at concentrations ranging from 50 to 1,000 μg/mL (*n* = 3, mean ± SD, *p < 0.05, **p < 0.01).

The effects of *G. frondosa* polysaccharide-derived fractions on RAW 264.7 macrophage proliferation and immune mediator production are shown in Figures 7, 8. The tested fractions increased RAW 264.7 macrophage proliferation in a concentration-dependent manner. At 500 μg/mL, they also increased NO production and TNF-α, IL-1β, and IL-6 levels compared with the untreated control.

Compared with CP, PP showed stronger effects on macrophage activation. Among the three PP fractions, PP-3 induced the greatest increases in macrophage proliferation and immune mediator production, showing the strongest macrophage-activation response under the present *in vitro* conditions. These findings suggest preliminary associations among molecular weight, compositional features, and cell-based activity, but they do not establish a definitive structure–activity relationship. Moreover, NO and cytokine production alone does not identify the receptor-mediated or intracellular signaling pathways involved. Because endotoxin-specific controls were not included, the possibility that trace endotoxin contamination contributed to macrophage activation cannot be excluded. In addition, because an LPS positive control was not included, the magnitude of macrophage activation induced by PP-3 could not be benchmarked against a standard macrophage-activating stimulus.

### 3.6 FITC labeling, iodination, and purification of PP-3

Based on the cell-based activity results, PP-3 was selected for *in vivo* biodistribution analysis. Because polysaccharides do not react directly with iodine, PP-3 was first labeled with FITC and then subjected to iodination. The resulting FITC-labeled and iodine-substituted product was designated PP-FI.

**Figure 9:**
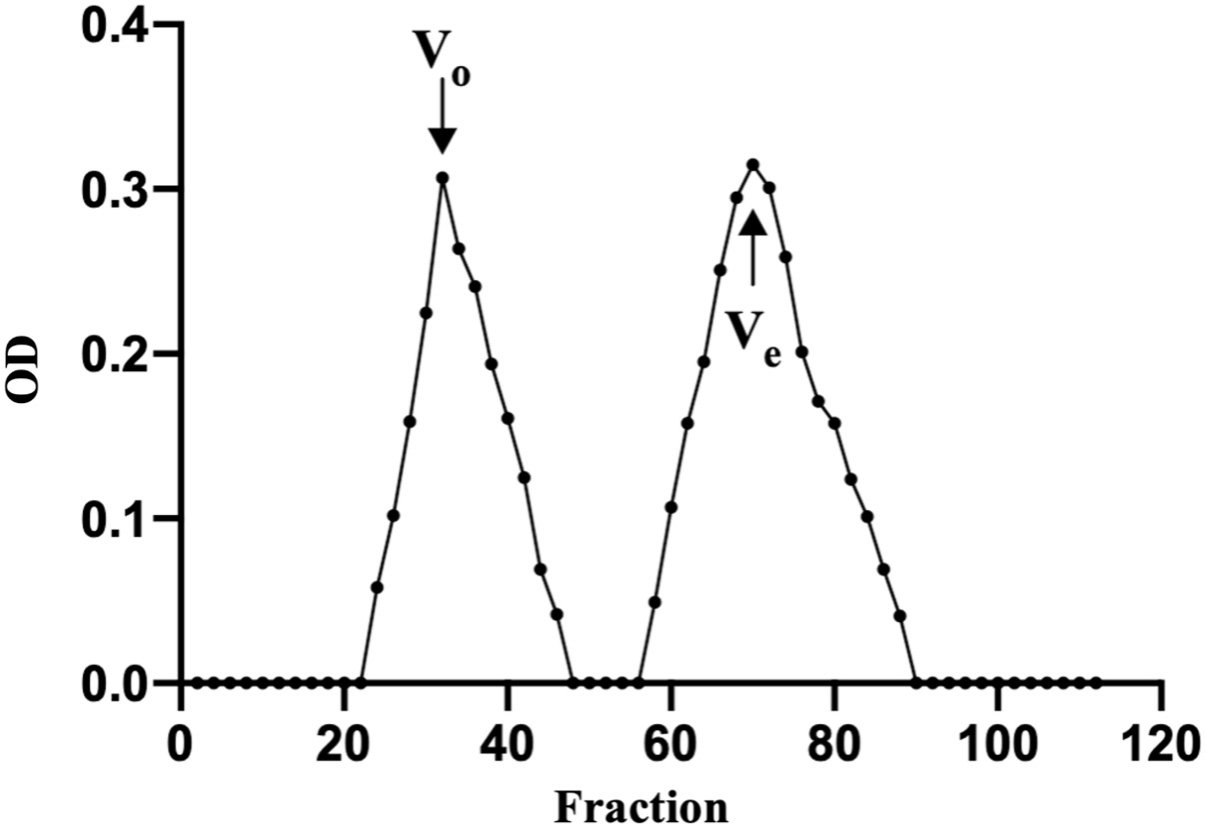
Purification of PP-FI using a Sephadex G-10 column.

To remove free iodine and small-molecule impurities, the reaction mixture was purified using Sephadex G-10 gel filtration. The elution profile is shown in Figure 9. Fractions 24–46 were positive by phenol-sulfuric acid colorimetric detection, and fractions 22–48 corresponding to PP-FI were collected, concentrated, and dried for subsequent analysis. Because ICP-MS detects iodine rather than the carbohydrate backbone itself, the measured signal should be interpreted as iodine associated with labeled PP-3-derived material. Therefore, subsequent biodistribution analysis of PP-FI provides preliminary tracer-based distribution information, but does not prove the distribution of intact native PP-3.

### 3.7 ICP-MS method validation and iodination rate of PP-FI

A NaI standard curve was generated in 1% TMAH using iodine concentrations of 1, 10, 50, 100, 200, and 300 ng/mL. The standard curve equation was *y* = 1.001*x* + 2.25, with a correlation coefficient of 0.9999, showing good linearity over the tested range.

ICP-MS method validation results are shown in Supplementary Tables S1–S4. The RSD for precision was 0.23% (Supplementary Table S1), and the RSD for sample stability over 24 h was 0.37% (Supplementary Table S2). Reproducibility testing showed an RSD of 0.58% (*n* = 6; Supplementary Table S3). Spike recovery ranged from 90% to 110%, with an RSD of 2.57% (Supplementary Table S4). The iodination rates of three independently prepared PP-FI samples were 2.49%, 2.50%, and 2.51% (Supplementary Table S5).

### 3.8 Biodistribution of PP-FI

The established ICP-MS standard curve was used to quantify iodine content in blood and organ samples. Blood iodine content and calculated PP-FI absorption rates are summarized in Table 4. After oral administration, the calculated absorption rate of PP-FI reached its highest value at 2 h (5.25%) and then decreased at 3 h.

**Table 4:** Iodine content and absorption rates of PP-FI in rat blood at different time points.

Time	*M* (ng/mL)	Cb (mg)	AR (%)
1 h	181.30 ± 2.10	6.71 ± 0.08	3.35 ± 0.04
2 h	283.87 ± 1.21	10.50 ± 0.04	5.25 ± 0.02
3 h	129.54 ± 1.95	4.79 ± 0.07	2.40 ± 0.04

Iodine-equivalent concentration and estimated whole-blood recovery of PP-FI-derived iodine signal at different time points. M, Iodine content measured via ICP-MS; Cb, PP-FI content in the blood; AR, Absorption rate of PP-FI in the blood.

At 3 h after administration, PP-FI distribution varied across organs (Table 5). The liver contained the highest amount of PP-FI (1.77 mg), followed by the kidney (1.01 mg). Lower amounts were detected in the heart (0.25 mg), spleen (0.17 mg), and lung (0.12 mg). These results show that orally administered PP-FI was mainly detected in the liver and kidney under the conditions of this experiment.

**Table 5:** Biodistribution of PP-FI in rat organs 3 h post-administration.

Organs	M (ng/mL)	Co (mg)
Liver	254.58 ± 2.22	1.77 ± 0.03
Heart	245.58 ± 2.87	0.25 ± 0.01
Spleen	219.48 ± 1.06	0.17 ± 0.01
Lung	199.49 ± 2.75	0.12 ± 0.01
Kidney	245.25 ± 1.65	1.01 ± 0.02

M, Iodine content measured via ICP-MS; Co, PP-FI content in organs.

## 4 Discussion

This study integrated molecular-weight fractionation, preliminary structural characterization, cell-based bioactivity assays, and ICP-MS-based biodistribution analysis to evaluate polysaccharide-derived fractions isolated from *G. frondosa*. The principal finding was that PP-3, the lowest-apparent-molecular-weight fraction (< 1 kDa), showed the highest total sugar content, a relatively simple glucose-rich methylation profile characterized by abundant terminal Glcp residues and limited internal glucose linkages, the strongest preliminary *in vitro* antiproliferative activity in S180 and HepG2 cells, and the greatest macrophage-activation response in RAW 264.7 cells among the tested fractions. *In vivo* biodistribution analysis further showed that orally administered iodine-labeled PP-3-derived material had limited systemic absorption and that iodine signal was mainly detected in the liver and kidney. Together, these findings suggest preliminary associations among molecular-weight distribution, compositional/linkage features, cell-based bioactivity, and labeled-material distribution behavior of *G. frondosa* polysaccharide-derived fractions.

Although monosaccharide composition and methylation analysis provided useful preliminary information on the compositional and linkage features of the separated fractions, the present study did not include NMR spectroscopy. Therefore, the proposed structural features, including the glucose-rich composition, abundant terminal Glcp residues, and detected internal glucose linkages in PP-3, should be regarded as preliminary rather than definitive. NMR-based structural elucidation will be required in future studies to confirm anomeric configuration, linkage positions, branching patterns, and possible higher-order conformational features. Accordingly, the present findings should be interpreted as preliminary structure-associated observations rather than a definitive structure–activity relationship.

The extremely low apparent molecular weight of PP-3 also requires cautious interpretation. An apparent value of 184 Da is close to the monosaccharide or very-low-oligosaccharide range and is not consistent with a conventional high-molecular-weight polysaccharide. In addition, this value was estimated by GPC using dextran calibration standards covering the 1–70 kDa range; therefore, the PP-3 value below 1 kDa should be considered an apparent estimate under the current chromatographic conditions rather than an exact absolute molecular mass. Based on ultrafiltration and GPC analysis, PP-3 is more appropriately defined as a low-molecular-weight carbohydrate/oligosaccharide-rich fraction derived from *G. frondosa* polysaccharides. The exact degree of polymerization and detailed chemical identity of PP-3 require further validation using additional structural methods, such as GPC with low-molecular-weight carbohydrate standards, LC-MS/MS, MALDI-TOF MS, and NMR spectroscopy. Therefore, PP-3 should not be interpreted as a structurally defined monosaccharide, disaccharide, or conventional polysaccharide based on the present GPC data alone.

A notable observation was that PP-3 showed stronger cell-based activity than the higher-molecular-weight fractions. This finding may appear different from many previous reports in which high-molecular-weight or particulate β-glucans exhibit stronger immunostimulatory effects. However, the activity of polysaccharide-derived fractions is not determined by molecular weight alone. Solubility, linkage pattern, branching degree, conformation, purity, residual protein content, and assay conditions may all influence biological response. In the present study, PP-3 had the highest sugar content and lower residual protein content than PP-1; however, residual protein was still detectable, and the possibility that protein-associated carbohydrates, glycoprotein-like components, or residual protein contributed to the observed bioactivity cannot be excluded. Therefore, the stronger activity of PP-3 should be interpreted as the activity of the isolated PP-3 fraction as a whole rather than as definitive evidence that the carbohydrate component alone was responsible. Its smaller apparent molecular size, glucose-rich composition, and simpler methylation profile may have contributed to differences in solubility or cellular interaction under the assay conditions used.

The macrophage-activation response induced by PP-3 should be interpreted cautiously because the present study measured functional activation markers but did not examine receptor engagement or downstream signaling pathways. Increased NO, TNF-α, IL-1β, and IL-6 production in RAW 264.7 macrophages indicates immune-cell activation under the present *in vitro* conditions, but these endpoints alone do not identify the molecular mechanisms responsible. Dectin-1, complement receptor 3, and other pattern-recognition receptors have been implicated in β-glucan-mediated immune activation, but receptor engagement can vary according to molecular weight, solubility, branching, linkage pattern, and particulate state. Goodridge et al. reported that Dectin-1 activation is strongly influenced by particulate β-glucan presentation, whereas soluble β-glucans may engage immune pathways differently (33). Han et al. also emphasized that the structure-function relationship of β-glucans depends on multiple physicochemical characteristics rather than molecular weight alone (34). Therefore, the relatively strong macrophage-activation response observed for PP-3 may be associated with its low apparent molecular weight, glucose-rich composition, methylation-inferred linkage features, and possible differences in solubility or cellular accessibility. However, this interpretation remains hypothetical, because receptor-blocking assays, pathway-inhibition experiments, and analyses of signaling molecules such as NF-κB or MAPK were not performed in the present study.

Another important consideration is the potential influence of endotoxin contamination on macrophage activation assays. RAW 264.7 macrophages are highly responsive to endotoxin, and trace endotoxin contamination may induce NO and pro-inflammatory cytokine production. In the present study, endotoxin-specific controls, such as Limulus amebocyte lysate (LAL) testing, endotoxin-removal validation, or polymyxin B neutralization, were not performed. Therefore, the observed increases in NO, TNF-α, IL-1β, and IL-6 should be interpreted as preliminary macrophage-activation responses of the isolated PP-3 fraction rather than definitive evidence of endotoxin-free polysaccharide-specific immune stimulation. Future studies should include endotoxin quantification, endotoxin-removal procedures, polymyxin B neutralization controls, and endotoxin-free experimental conditions to clarify the contribution of the carbohydrate fraction itself to macrophage activation.

The absence of positive controls also limits interpretation of the biological relevance and relative potency of the observed responses. In macrophage assays, lipopolysaccharide (LPS) is commonly used as a positive control to benchmark macrophage activation, whereas doxorubicin or cisplatin can serve as reference cytotoxic agents in cancer-cell assays. These controls were not included in the present study. Therefore, the effects of PP-3 should be interpreted as preliminary cell-based responses under the present screening conditions rather than as evidence of potency comparable to established immune stimuli or antitumor agents. Future studies should include appropriate positive controls to contextualize the magnitude and biological relevance of the observed macrophage-activation and antiproliferative effects.

The antiproliferative findings should also be interpreted cautiously. PP-3 showed the strongest inhibitory activity against S180 and HepG2 cells among the tested fractions in the CCK-8 cell-viability assay. These results support PP-3 as the most active fraction in the present *in vitro* screening assays, but they do not establish selective antitumor activity or direct therapeutic efficacy *in vivo*. The concentrations used in the cell assays were selected for comparative screening of polysaccharide-derived fractions and are relatively high. Moreover, given the limited oral absorption observed in this study, these concentrations may not reflect systemically achievable levels after oral administration. Because positive antitumor controls such as doxorubicin or cisplatin and normal non-tumor cell controls were not included, the relative potency and cancer-cell selectivity of PP-3 could not be determined. Therefore, the antiproliferative results should be regarded as preliminary evidence of relative cell-based bioactivity rather than proof of selective anticancer potential. Future studies should include normal-cell controls, selectivity-index assessment, dose–response modeling, gastrointestinal cell models such as Caco-2 cells, and *in vivo* efficacy and safety evaluation.

The biodistribution component is an important methodological feature of this study, but its interpretation requires caution. Natural polysaccharides are difficult to quantify *in vivo* because they lack strong intrinsic signals and may be structurally similar to endogenous carbohydrates. By combining FITC labeling, iodination, and ICP-MS detection, we established a non-radioactive approach for tracing iodine-labeled PP-3-derived material in blood and organs. However, FITC labeling and iodination are chemical modifications that may alter the original physicochemical properties of PP-3, including molecular size, hydrophilicity/hydrophobicity, charge distribution, conformation, absorption behavior, tissue distribution, and possibly biological activity. Therefore, the biodistribution findings should not be interpreted as a full pharmacokinetic profile of the native PP-3 fraction. Importantly, the labeled PP-FI was used only for biodistribution analysis, whereas all cell-based bioactivity assays were performed using native, unlabeled polysaccharide-derived fractions. Another important consideration is that ICP-MS quantifies iodine signal rather than the intact carbohydrate structure. Therefore, the detected iodine signal cannot distinguish intact labeled PP-3 from iodine-containing fragments, metabolites, or degradation products generated after administration. The observed blood and tissue signals should therefore be interpreted as preliminary distribution signals of iodine-labeled PP-3-derived material, rather than definitive evidence for intact polysaccharide distribution. Future studies combining radiolabeling, fluorescent imaging, chromatographic separation, molecular-weight tracking, or mass spectrometry-based structural analysis would be needed to confirm the integrity and fate of PP-3 after administration.

The *in vivo* results showed that PP-FI had a low calculated absorption rate after oral administration, peaking at 2 h and decreasing by 3 h. Organ analysis showed preferential detection in the liver and kidney. This pattern is consistent with previous studies of other mushroom-derived polysaccharides, including polysaccharides from *Ganoderma lucidum* and *Lentinus edodes*, which have also shown low oral bioavailability and preferential liver and/or kidney distribution (35–37). The liver signal may reflect hepatic uptake or metabolism, whereas kidney detection may be related to the small molecular size of PP-3 and renal handling. Previous studies of natural polysaccharides have similarly suggested that molecular weight and spatial structure can influence absorption, distribution, and renal accumulation (38, 39).

This study has several strengths. First, it evaluated *G. frondosa* polysaccharides after molecular-weight fractionation rather than relying only on crude extracts. Second, it linked compositional and structural characterization with both antiproliferative and immunostimulatory assays. Third, it introduced an ICP-MS-based non-radioactive tracing strategy for preliminary *in vivo* biodistribution analysis. These features make the study relevant to natural-product characterization and functional-food development.

From a broader application perspective, the development of bioactive fractions from edible mushrooms is consistent with increasing interest in sustainable utilization of natural bioresources and value-added food processing. Tang et al. emphasized that natural agricultural resources can serve as multifunctional platforms for food, nutraceutical, cosmetic, and pharmaceutical applications, supporting the concept of converting bioresources into higher-value functional products (40). Bushueva et al. (41) also highlighted the socioeconomic and environmental relevance of food processing sectors, underscoring the importance of developing value-added bioactive ingredients from natural resources. In this context, molecular-weight fractionation and preliminary bioactivity screening of *G. frondosa* polysaccharide-derived fractions may contribute to the development of mushroom-derived functional-food ingredients.

Several limitations should be acknowledged. First, although monosaccharide composition, apparent molecular-weight distribution, and methylation analysis were used to characterize the separated fractions, NMR spectroscopy was not performed. Therefore, the detailed structure of PP-3, including anomeric configuration, linkage positions, branching features, and higher-order conformation, remains to be confirmed. Second, because PP-3 showed an extremely low apparent molecular weight, it should be interpreted as a low-molecular-weight carbohydrate/oligosaccharide-rich fraction rather than a conventional high-molecular-weight polysaccharide. The exact degree of polymerization of PP-3 could not be determined from the current GPC analysis alone, and future studies should validate this fraction using methods suitable for low-molecular-weight carbohydrates or oligosaccharides. Third, although purification increased the total sugar content and reduced protein content, residual protein was still detected in the separated fractions; therefore, the possibility that protein-associated carbohydrates, glycoprotein-like components, or residual protein contributed to the observed bioactivity cannot be excluded. Future studies should include more stringent deproteinization, protease-digestion controls, glycoprotein-specific characterization, and comparison of bioactivity before and after protein removal to clarify the respective contributions of carbohydrate and protein-associated components. Fourth, all fractions were prepared from a single authenticated bulk lot of *G. frondosa*, and batch-to-batch variability was not evaluated. Fifth, the antiproliferative assays were performed only *in vitro* and at relatively high concentrations, and no *in vivo* antitumor efficacy model was included. Because normal non-tumor cell controls and positive antitumor controls such as doxorubicin or cisplatin were not included, the selectivity and relative potency of PP-3 toward cancer cells could not be determined. Sixth, the immunostimulatory mechanism was inferred from macrophage activation markers and literature-based receptor biology rather than confirmed by receptor-blocking assays, pathway-inhibition experiments, or downstream signaling analyses. In addition, endotoxin contamination was not directly assessed using LAL testing, endotoxin-removal validation, or polymyxin B neutralization, and an LPS positive control was not included; therefore, the observed increases in NO and cytokine production should be interpreted as preliminary macrophage-activation responses of the isolated PP-3 fraction rather than definitive evidence of endotoxin-free polysaccharide-specific immune stimulation or activity comparable to a standard macrophage-activating stimulus. Future studies should include endotoxin quantification, neutralization controls and appropriate positive controls to determine whether macrophage activation is attributable to the carbohydrate fraction itself. Seventh, although the FITC-iodine labeling strategy enabled ICP-MS detection, chemical labeling may alter the molecular size, hydrophilicity/hydrophobicity, charge distribution, native conformation, absorption behavior, biological activity, or tissue distribution of PP-3. Moreover, ICP-MS detects iodine signal and cannot distinguish intact labeled PP-3 from labeled fragments, metabolites, or iodine-containing degradation products. Therefore, the biodistribution findings should be interpreted as preliminary distribution data for iodine-labeled PP-3-derived material rather than a full pharmacokinetic profile of the native PP-3 fraction.

In summary, PP-3 was identified as the low-molecular-weight carbohydrate/oligosaccharide-rich fraction with the strongest preliminary cell-based activity among the tested *G. frondosa* polysaccharide-derived fractions in the present study. Its activity may be associated with its high sugar content, glucose-rich composition, low apparent molecular weight, simple methylation profile, and possible differences in solubility or cellular accessibility. However, because NMR spectroscopy, degree-of-polymerization analysis, endotoxin testing, and protein-removal controls were not performed, these findings should be interpreted as preliminary structure-associated observations rather than definitive mechanistic or structure–activity evidence. The ICP-MS-based biodistribution results should also be interpreted cautiously: they suggest limited oral absorption of iodine-labeled PP-3-derived material and preferential detection of iodine signal in the liver and kidney, but do not establish a full pharmacokinetic profile or intact-tissue distribution of the native PP-3 fraction. Future studies should validate batch reproducibility, confirm detailed structural features, clarify receptor-mediated and endotoxin-independent mechanisms, evaluate gastrointestinal and *in vivo* models, and develop formulation strategies to improve oral delivery or local functional effects.

## 5 Conclusion

In this study, polysaccharides were extracted and purified from *G. frondosa* and then separated into three polysaccharide-derived molecular-weight fractions. Among them, PP-3 was interpreted as a low-molecular-weight carbohydrate/oligosaccharide-rich fraction and showed the highest total sugar content, a relatively simple glucose-rich methylation profile characterized by abundant terminal Glcp residues and limited internal glucose linkages, and the strongest preliminary cell-based bioactivity in the present screening assays, including inhibitory effects on S180 and HepG2 cell viability and macrophage-activation responses in RAW 264.7 cells. However, the antiproliferative findings should be interpreted cautiously because normal non-tumor cell controls and *in vivo* efficacy models were not included, and selective cytotoxicity toward cancer cells could not be determined. Because NMR spectroscopy was not performed, these structural features should be regarded as preliminary and require further confirmation. These findings suggest that low apparent molecular weight and related compositional/linkage features may be associated with the bioactivity of *G. frondosa* polysaccharide-derived fractions, although the possible contribution of residual protein or glycoprotein-like components cannot be excluded.

This study also established an FITC-assisted iodination and ICP-MS-based approach for preliminary biodistribution analysis of iodine-labeled PP-3-derived material. Orally administered PP-FI showed limited systemic absorption, and iodine signal was mainly detected in the liver and kidney; however, these findings should be interpreted as preliminary tracer-based distribution data for iodine-labeled PP-3-derived material rather than as a full pharmacokinetic profile or intact-tissue distribution of the native PP-3 fraction. Overall, these results identify PP-3 as a low-molecular-weight carbohydrate/oligosaccharide-rich fraction with preliminary cell-based bioactivity among the tested *G. frondosa* polysaccharide-derived fractions and support further investigation of mushroom-derived polysaccharide-derived fractions for functional-food and natural-product applications.

## Data Availability

The original contributions presented in the study are included in the article/Supplementary material, further inquiries can be directed to the corresponding authors.
