# Supplementary material for: Molecular-weight fractionation, preliminary structural characterization, bioactivity, and ICP-MS-based biodistribution of Grifola frondosa polysaccharide-derived fractions
Source: Front Nutr. 2026 Aug 27;13:1930891. doi: 10.3389/fnut.2026.1930891 (PMC13558435; doi:10.3389/fnut.2026.1930891)
Supplement: Supplementary file 1 [file Supplementary_file_1.docx]

Supplementary Material

Fig. S1. Molecular weight (MW) distribution of polysaccharide fractions (A) PP-1, (B) PP-2, and (C) PP-3, analyzed by gel permeation chromatography (GPC).

Table S1. Precision Determination of PP-FI using ICP-MS.

| Number | 1 | 2 | 3 | 4 | 5 | 6 | RSD (%) |
| --- | --- | --- | --- | --- | --- | --- | --- |
| Measured Value (ng/mL) | 199.86 | 200.74 | 200.46 | 199.84 | 200.97 | 200.38 | 0.23 |

Table S2. Stability determination of PP-FI over 24 hours.

| Time (hour) | 0 | 2 | 16 | 24 | RSD (%) |
| --- | --- | --- | --- | --- | --- |
| Measured Value (ng/mL) | 199.17 | 200.84 | 200.37 | 199.64 | 0.37 |

Table S3. Reproducibility of PP-FI using ICP-MS.

| Number | 1 | 2 | 3 | 4 | 5 | 6 | Mean | RSD (%) |
| --- | --- | --- | --- | --- | --- | --- | --- | --- |
| Measured Value (ng/mL) | 199.37 | 199.46 | 200.85 | 202.48 | 201.36 | 200.57 | 200.68 | 0.58 |

Table S4. Recovery Rate Determination of PP-FI using ICP-MS.

| Number | Iodine Concentration  (ng/mL) | Spike Amount (ng/mL) | Measured Value (ng/mL) | Recovery Rate (%) |
| --- | --- | --- | --- | --- |
| 1 | 100.20 | 103.48 | 199.48 | 95.94 |
| 2 | 100.20 | 105.28 | 208.57 | 102.94 |
| 3 | 100.20 | 101.42 | 200.97 | 99.36 |
| 4 | 100.20 | 102.44 | 204.33 | 101.65 |
| 5 | 100.20 | 100.12 | 198.84 | 98.52 |
| 6 | 100.20 | 102.44 | 200.47 | 97.88 |

Table S5. Iodination rates of three independently prepared PP-FI samples.

| Batch | 1 | 2 | 3 | Mean |
| --- | --- | --- | --- | --- |
| Measured Value (ng/mL) | 199.58 | 200.58 | 200.89 | 200.35 |
| Iodination Rate (%) | 2.49 | 2.50 | 2.51 | 2.50 |
